# Osmotic response in *Leptospirillum ferriphilum* isolated from an industrial copper bioleaching environment to sulfate

**DOI:** 10.3389/fmicb.2024.1369244

**Published:** 2024-05-24

**Authors:** Dayana Arias, Víctor Zepeda, Ivan Nancucheo, Manuel Saldaña, Pedro A. Galleguillos

**Affiliations:** ^1^Laboratory of Molecular Biology and Applied Microbiology, Centro de Investigación en Fisiología y Medicina de Altura (FIMEDALT), Departamento Biomédico, Facultad de Ciencias de la Salud, Universidad de Antofagasta, Antofagasta, Chile; ^2^Scientific and Technological Research Centre for Mining Research, CICITEM, Antofagasta, Chile; ^3^Facultad de Ingeniería, Arquitectura y Diseño, Universidad San Sebastián, Concepción, Chile; ^4^Faculty of Engineering and Architecture, Arturo Prat University, Iquique, Chile

**Keywords:** bioleaching, compatible solutes, *Lesptospirillum ferriphilum*, osmotic strength, sulfate

## Abstract

Iron and sulfur-oxidizing microorganisms play important roles in several natural and industrial processes. *Leptospirillum* (*L.*) *ferriphilum*, is an iron-oxidizing microorganism with a remarkable adaptability to thrive in extreme acidic environments, including heap bioleaching processes, acid mine drainage (AMD) and natural acidic water. A strain of *L. ferriphilum* (IESL25) was isolated from an industrial bioleaching process in northern Chile. This strain was challenged to grow at increasing concentrations of sulfate in order to assess changes in protein expression profiles, cells shape and to determine potential compatible solute molecules. The results unveiled changes in three proteins: succinyl CoA (SCoA) synthetase, isocitrate dehydrogenase (IDH) and aspartate semialdehyde dehydrogenase (ASD); which were notably overexpressed when the strain grew at elevated concentrations of sulfate. ASD plays a pivotal role in the synthesis of the compatible solute ectoine, which was identified along with hydroxyectoine by using matrix-assisted laser desorption/ionization-time of flight mass spectrometry (MALDI-TOF). The relationship between IDH, SCoA, and ectoine production could be due to the TCA cycle, in which both enzymes produce metabolites that can be utilized as precursors or intermediates in the biosynthesis of ectoine. In addition, distinct filamentous cellular morphology in *L. ferriphilum* IESL25 was observed when growing under sulfate stress conditions. This study highlights a new insight into the possible cellular responses of *L. ferriphilum* under the presence of high sulfate levels, commonly found in bioleaching of sulfide minerals or AMD environments.

## Graphical Abstract

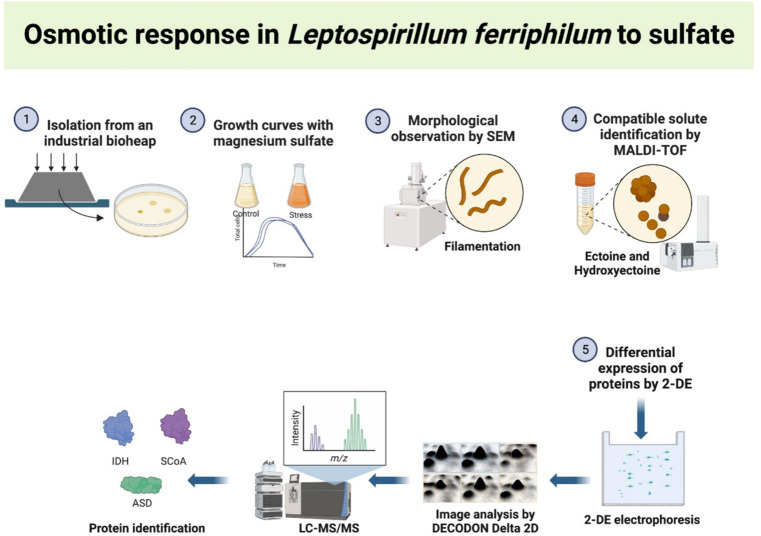

## 1 Introduction

The osmotic strength in extremely acidic water bodies can vary in a wide range, mainly due to the concentration of dissolved inorganic solutes. Mine process water under acid-generating environments can contain up to hundreds grams of sulfate per liter, originated from the oxidative dissolution of pyrite and other sulfide minerals, which is catalyzed by species of acidophilic iron and sulfur-oxidizing microorganisms (Masindi et al., 2019; Rambabu et al., 2021; Hernández et al., 2022). Among the different anions, high concentrations of sulfate (from 110 to 760 g/L) have been detected in extremely acidic water bodies (pH from −3.60 to +1.51) including ponds and drainage streams within the abandoned Richmond mine at Iron Mountain, California (Nordstrom et al., 2000). A pond adjacent to a dump at the abandoned São Domingos copper mine in Portugal was reported to display seasonal changes in concentrations of sulfate (ranging between 14–570 g/L) and pH (between 0.13–2.55) (Ferreira et al., 2021). Extremophilic bacteria and archaea, including the iron-oxidizing autotroph *Lesptospirillum ferriphilum* and the iron-oxidizing heterotrophs *Ferroplasma acidarmanus* and *Ferrimicrobium acidiphilum* have been isolated from sites with high osmotic strengths and low pH environments (Hedrich and Schippers, 2020).

Similar conditions, combining extreme acidity and high osmotic strength can be found in heap bioleaching operations because of the dissolution of minerals under the extremely acidic and oxidizing conditions used to extract metals from sulfidic ores (Hedrich and Schippers, 2020). In addition, bioleaching operations located in desert areas optimize the usage of water by constantly recirculating the solutions in the industrial circuit and thus, increasing the concentration of inorganic compounds and therefore the osmotic strength.

Among all species of acidophilic prokaryotes identified in heap bioleaching operations, the most widely reported are members of *Acidithiobacillus*, *Leptospirillum*, *Sulfobacillus*, *Ferroplasma* and *Acidiphilium* genera (Demergasso et al., 2005; Rawlings and Johnson, 2007; Schippers et al., 2014; Jia et al., 2021). These species have shown different osmo-tolerance level to osmotic strength produced by increasing chloride ions, thus their tolerance to sodium chloride (halo-tolerance) varies widely, with some of the “primary” (iron-oxidizing autotrophic) species being far less tolerant to chloride that some sulfur-oxidizers and heterotrophic species (Boxall et al., 2017; Corbett et al., 2022). Previous studies have shown *Leptospirillum* spp. as one of the most abundant iron oxidizer species found in natural acidic environments and copper heap bioleaching processes, when sulfate reaches concentrations about 1.0 M or even higher (Demergasso et al., 2005; Diaby et al., 2007; Moreno-Paz et al., 2010; Boxall et al., 2017; Martínez et al., 2022); however, adaptation of *Leptospirillum* spp. to high osmotic potentials is poorly understood.

While extensive research has been conducted on the effects of sodium chloride and potassium chloride as models of salt; to our knowledge little attention has been given to the influence of elevated osmotic strength produced by sulfate on iron-oxidizing bacteria. This study aims to bridge this knowledge gap by investigating the cellular responses of the acidophilic iron oxidizing bacteria *L. ferriphilum* IESL25, isolated from an industrial heap bioleaching operation located in the Atacama Desert (northern Chile), when exposed to varying concentrations of sulfate, mimicking in part the evolution of the liquid phase in the heap industrial bioleaching process. Our comprehensive analysis encompasses assessments on cell growth, morphological changes, alterations in protein expression patterns, and the production of osmoprotectants. This research provides valuable insights into the cellular adaptations and stress responses under high sulfate concentrations, highlighting the implications in the bioleaching of sulfide minerals at industrial scale.

## 2 Materials and methods

### 2.1 Isolation of *Lesptospirillum ferriphilum* IESL25

*L. ferriphilum* strain IESL25 was isolated from an industrial bioheap process located in northern Chile. For this, pregnant leach solution (PLS, with 30 g/L sulfate concentration) containing microorganisms and dissolved copper from the heap ore was directly streaked onto iron overlay plates containing autotrophic basal salt, trace elements (ABS/TE); (Nancucheo et al., 2016) and ferrous iron incubated at 37°C for 15 days under oxic conditions, as previously described by Rawlings et al. (2007). After the incubation period, colonies with deposition of red-brownish coloration were transferred into corresponding liquid media containing 50 mM ferrous iron, supplemented with ABS/TE pH 1.7 and incubated under same conditions (37°C for 15 days). Liquid cultures were scaled up to 50 mL and DNA was extracted for PCR-cloning and sequencing of 16S rRNA gene by vector cloning, as described previously (Okibe et al., 2003). The resulting sequence data were visualized using Chromas Lite version 2.01 and compared with gene sequences deposited in NCBI GeneBank database,^1^ using the nucleotide BLAST tool. ARB software^2^ was used in order to determine phylogenetic affiliation of the strain based on 16S rRNA sequence (Supplementary Figure S2). The 16S rRNA gene sequence of *L. ferriphilum* IESL25 is available from NCBI nucleotide database (Accession Number HQ902070).

### 2.2 Growth of *Lesptospirillum ferriphilum* IESL25 at increasing concentrations of sulfate

*L. ferriphilum* IESL25 was grown in ABS/TE medium supplemented with increasing concentrations of magnesium sulfate (MgSO_4_∙7H_2_O, Merck) and using an inoculum equivalent to 5% of the total culture volume. Since the culture medium contains sulfate salts, total sulfates concentration was analyzed by analytical gravimetric analysis, yielding total sulfate contents of 8 g/L for the control and 40, 80, and 100 g/L for the experimental cultures. Bacterial growth was conducted in triplicate and followed by microscopy using a Neubauer cell counting chamber and a Leyca MDLS microscope equipped for fluorescence and contrast phase techniques. DAPI staining technique was also used for cells counting at the lag and early exponential growth phases. Iron oxidation by *L. ferriphilum* IESL25 during the growth experiments was determined by periodical titration of ferrous iron with potassium dichromate (Vogel, 1961).

### 2.3 Cell morphology by scanning electron microscopy

Cultures containing cells grown in the presence of 8 g/L (control) and 80 g/L of sulfate were first fixed in 4% (v/v) glutaraldehyde (Fluka). Then, one milliliter of culture containing fixed cell samples was filtered through 0.22 μm pore size polycarbonate filtration membranes (Millipore). The cells were dehydrated by immersing the filter membranes for one minute in ethanol at increasing concentrations (20, 40, 60, 80, and 100%). Subsequently, the filters were prepared for microscopic observation by standard procedures including CO_2_ critical drying and gold coating. Finally, the prepared filters were observed in a JEOL JSM 6360LV scanning electronic microscope, at acceleration voltages ranging from 10 to 20 kV.

### 2.4 Identification of compatible solutes using hypo-osmotic shock approach and matrix-assisted laser desorption/ionization-time of flight mass spectrometry

A matrix-assisted laser desorption/ionization-time of flight mass spectrometry (MALDI-TOF) equipment (Bruker Daltonics, Germany) was used to analyze small molecular weight compounds released by bacteria subjected to hypo-osmotic shock approach (HSA). Briefly, for hypoosmotic shock approach, cells from cultures grown at different osmotic strength were harvested by centrifugation and cells pellet was exposed to low osmotic strength by resuspending in 5.0 mL of acidified MilliQ-grade water (pH 1.7, adjusted with sulfuric acid) for 10 min at 37°C. Then, bacterial debris were removed by centrifugation and the supernatants were filtered through 0.2 μm pore-sized membranes obtaining the extract. Each Extract was later mixed either with TiO_2_ (10 mg/mL) or CsI (30 mg/mL) as matrix, in a 1:1 ratio and spotted onto a target plate MTP384 (Bruker Daltonics, Germany). The spectral peaks obtained from extracts control (8 g/L) and high sulfate culture (80 g/L) were compared to identify differential mass/charge peaks. Each differential peak detected was identified by comparing with those of known common organic osmolytes produced by prokaryotes.

### 2.5 Two-dimensional electrophoresis

Protein extracts were prepared from *L. ferriphilum* cells harvested at exponential phase of growth (8 and 80 g/L of sulfate), according to a modified methodology reported by Li et al. (2009). Cells were harvested by centrifugation (1 L culture, at 10,000 g, 20 min), the mass of each cell pellet was estimated after centrifugation using the weight of the empty microcentrifuge tube and the weight of the same tube containing the pellet. The tubes containing the pellets were subjected to sonication in ice bath (6 times, 60% amplitude, constant 30 s) in buffer UTCTEDP (6 M Urea, 2 M Thiourea; 4% CHAPS; 2% Trirón X-100; 40 mM Tris; 1 mM EDTA; 5 mM MgCl2; 60 mM DTT; 1 mM PMSF, Protease inhibitors Roche, cOmplete^™^, Mini Protease Inhibitor Cocktail and 100 mg/L DNAse I). Later, the salts were removed using purification centrifugal filters (Amicon Ultra-0.5, Millipore cat. UFC500308), followed by a double precipitation using 5% TCA/Acetone. Protein final concentration was determined by using PierceTM Coomassie plus kit (Thermo Fisher cat. 23236) or 2D Quant kit (Cytiva 80-6483-56).

For the isoelectric focusing (IEF), 17 cm Immobilized pH Gradient (IPG) strips with a non-linear pH range of 5–8 (Bio-Rad) were used. These strips were rehydrated overnight with protein samples previously extracted and quantified using a rehydration buffer [6 M Urea; 2 M thiourea; 4% CHAPS; 0.27% (v/v) ampholytes 3–10; 0.13% (v/v) ampholytes 5–8, and 0.001% (w/v)]. The IEF process involved the following steps: an initial voltage of 500 V for 1 h, followed by an increase to 1,000 V for another hour, with a gradual ramp-up to 8,000 V, maintaining this voltage for a total of 32,000 volt-hours. These procedures were carried out in a Bio-Rad system at a constant temperature of 20°C. Subsequently, the rehydrated strips were equilibrated first with 0.5% Dithiothreitol (DTT), followed by re-equilibration in a buffer containing 4.5% iodoacetamide, which replaced DTT. Each equilibration step took 20 min. The second-dimension electrophoresis was conducted at a constant voltage of 100 V for 18 h.

Delta 2D 4.0 imaging software (Decodon, Greifswald, Germany) was used to perform gel image densitometric analysis. To create a unified master gel, the acquired gel images were merged, and common spots were identified and adjusted across all images. Normalization was then carried out based on the total spot density. Statistical analysis was conducted using the Delta 2D-based student’s *t*-test to assess the significance and statistical variations in protein expression. A *p*-value less than 0.05 was considered to indicate statistical significance. Only those spots exhibiting a significant differential level between control and high sulfate concentration were selected for identification purposes. The selected spots were excised of the gel and separately placed in tubes previously washed with Methanol (Merck) or Acetonitrile (Merck) and washed twice for 10 min with 50% Acetonitrile/Water (v/v). The tubes containing gel pieces were stored at −20°C until they were submitted to liquid chromatography coupled to tandem mass spectrometry (LC-MS/MS) analyses.

### 2.6 Protein identification by liquid chromatography coupled to tandem mass spectrometry

The partial prediction of the sequence of the proteins digested with trypsin was performed by LC-MS/MS with a 3D ion trap spectrometer (Thermo Electron). After the LC-MS/MS analyses, mass spectra were compared with the theoretical mass spectra against a *Leptospirillum* database using the SEQUEST program.^3^ Subsequently, the statistical program ProteinProphet^4^ was used for data filtering. This whole protein prediction procedure was performed at the Proteomic Services of the Cancer Center of the University of Maryland, United States, and is described in greater detail by Matthiesen and Bunkenborg (2020). Using the peptide sequences from each protein, a search was carried out with the NCBI BLASTp program (see text footnote 1) to identify the protein (Altschul et al., 1997). The Compute pI/Mw tool^5^ was used to obtain the theoretical isoelectric point and molecular size values.

## 3 Results and discussion

### 3.1 Isolation and 16S rRNA gene sequencing

**Figure 1:**
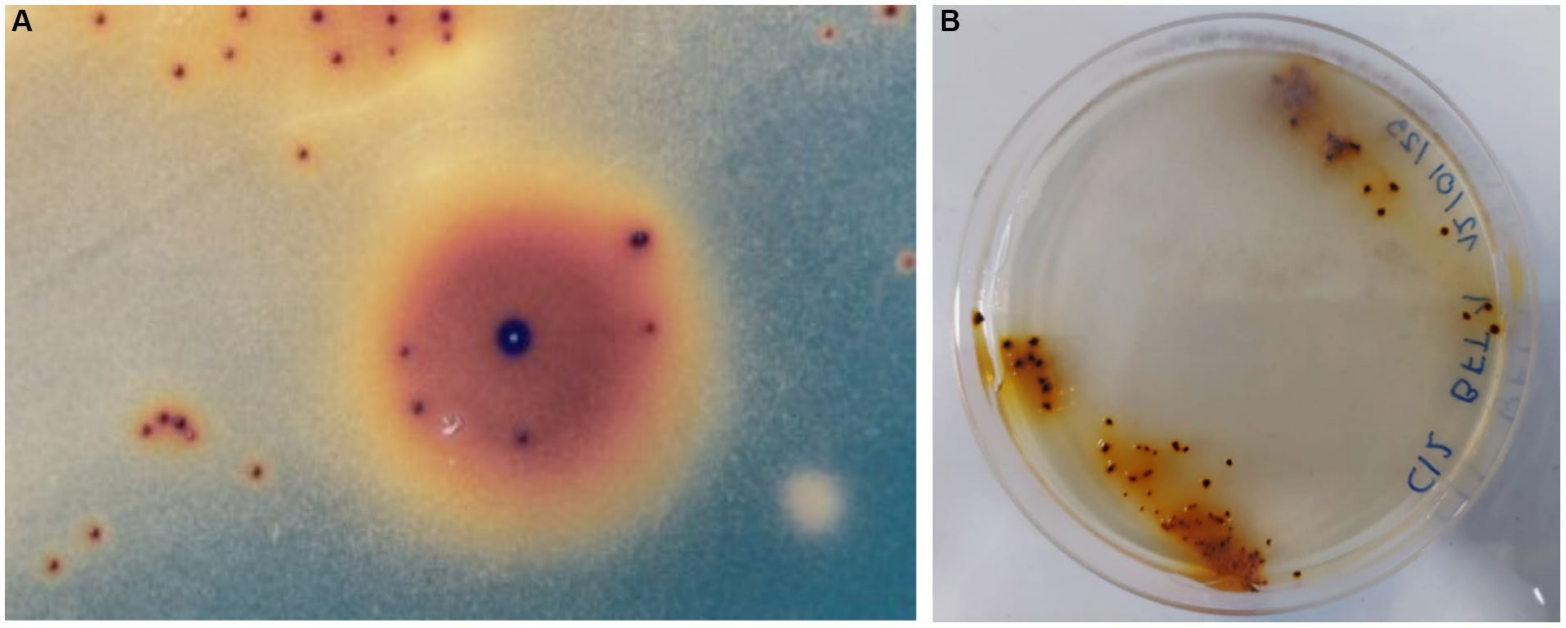
Autotrophic iron oxidized colonies growing onto overlay plates obtained from Minera Escondida copper bioleaching plant **(A)**. Bacterial colonies of *L. ferriphilum* IESL 25 **(B)**.

After an incubation period (between 15–20 days), several colonies of autotrophic iron oxidizers (red-brownish colour and between 0.2–0.5 mm) were obtained by directly streaking PLS onto overlay plates (Figure 1A). Figure 1B shows a second stage where a single colony was transferred an overlay plate. The partial 16S rRNA sequence of the isolate has been identified and assigned the name *L. ferriphilum* IESL25. This sequence has been deposited in the NCBI GenBank nucleotide database (accession number HQ902070).

### 3.2 Growth of *Leptospirillum ferriphilum* at increasing concentrations of sulfate and collection of cells for proteomic analysis

**Figure 2:**
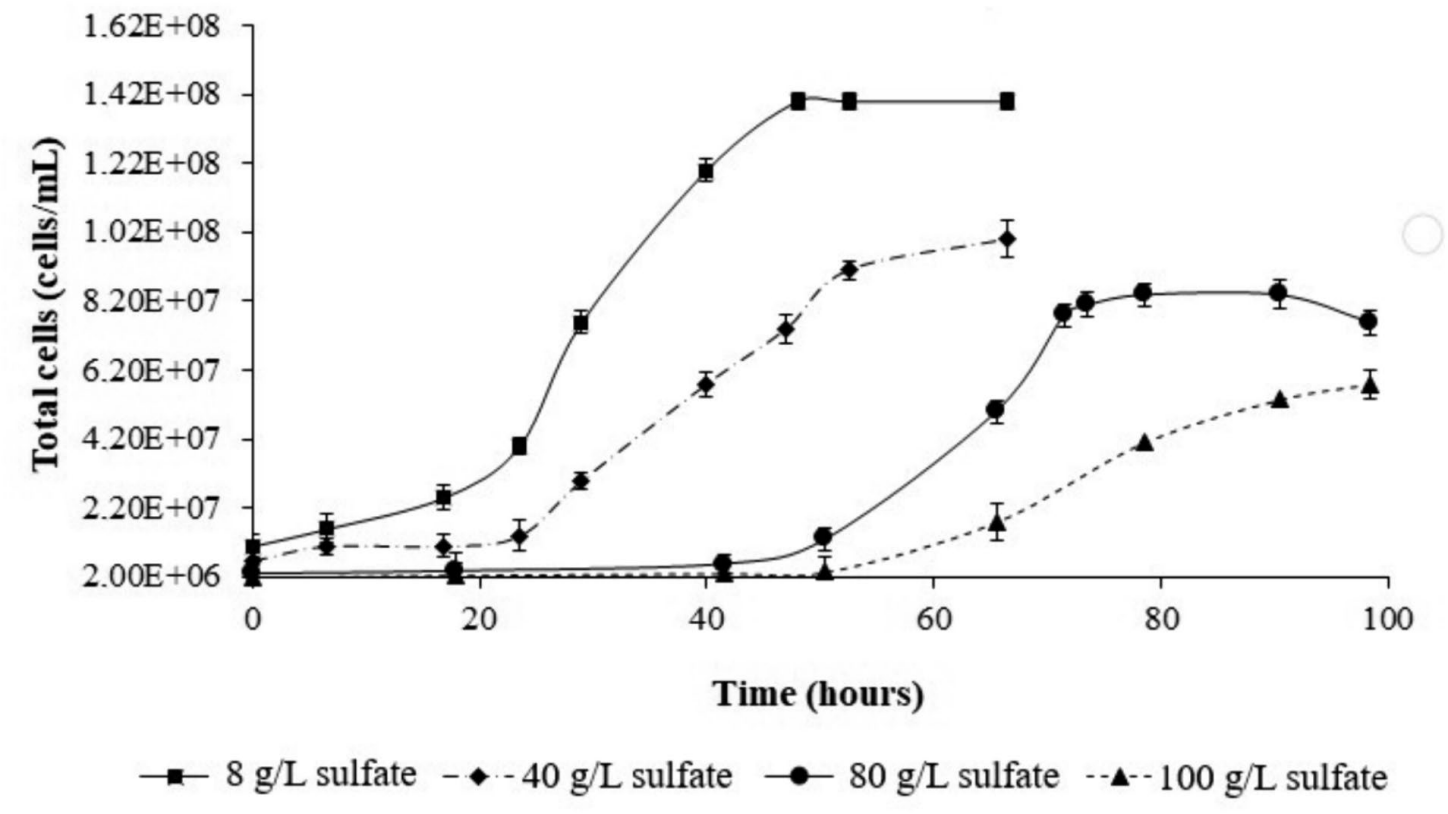
Growth curves of *L. ferriphilum* IESL 25 at different sulfate concentrations.

The effect of increasing sulfate on culture growth of *L. ferriphilum* IESL25 is shown in Figure 2. Initially, the isolate was grown in ferrous iron-ABS/TE medium containing 8 g/L of sulfate concentration and 50 mM of ferrous iron, obtaining a culture doubling time of 10.8 h. In the presence of higher sulfate concentration, the culture growth profile was affected producing a longer lag phase with higher doubling times. The doubling times in the presence of 40 g/L, 80 g/L and 100 g/L of sulfate were 11.0 h, 13.5 h, and 14.2 h, respectively. For proteomic analyses, the cells were harvested in the exponential growth phase, at the point when 90% of ferrous iron was oxidized to obtain the highest number of active cells. The harvesting points (90% of oxidized Fe^2+^) occurred at approximately 40, 60, and 75 h for the control condition culture, and in the presence of 80 g/L and 100 g/L of sulfate, respectively.

Based on the observations of growth profiles, it can be deduced that sulfate exerts a suppressive influence on the growth of *L. ferriphilum* IESL25 as the concentration increases. In the range of sulfate used in the experiments (8–100 g/L), the doubling time values increased following a linear correlation. In light of this information, it is evident that growth inhibition occurs, as indicated by a 24% increase in doubling time between the control condition and the highest sulfate concentration used in this study (100 g/L). Regarding industrial conditions, a previous report using acidophilic culture to recycle water from a low-grade ore heap process indicated between 110 and 130 g/L as the that the upper limits of tolerance by microorganisms to sulfate (Adaos, 2013).

### 3.3 Morphology of *Leptospirillum ferriphilum* ISL25 under control condition (8 g/L of sulfate) and osmotic stress (80 g/L)

**Figure 3:**
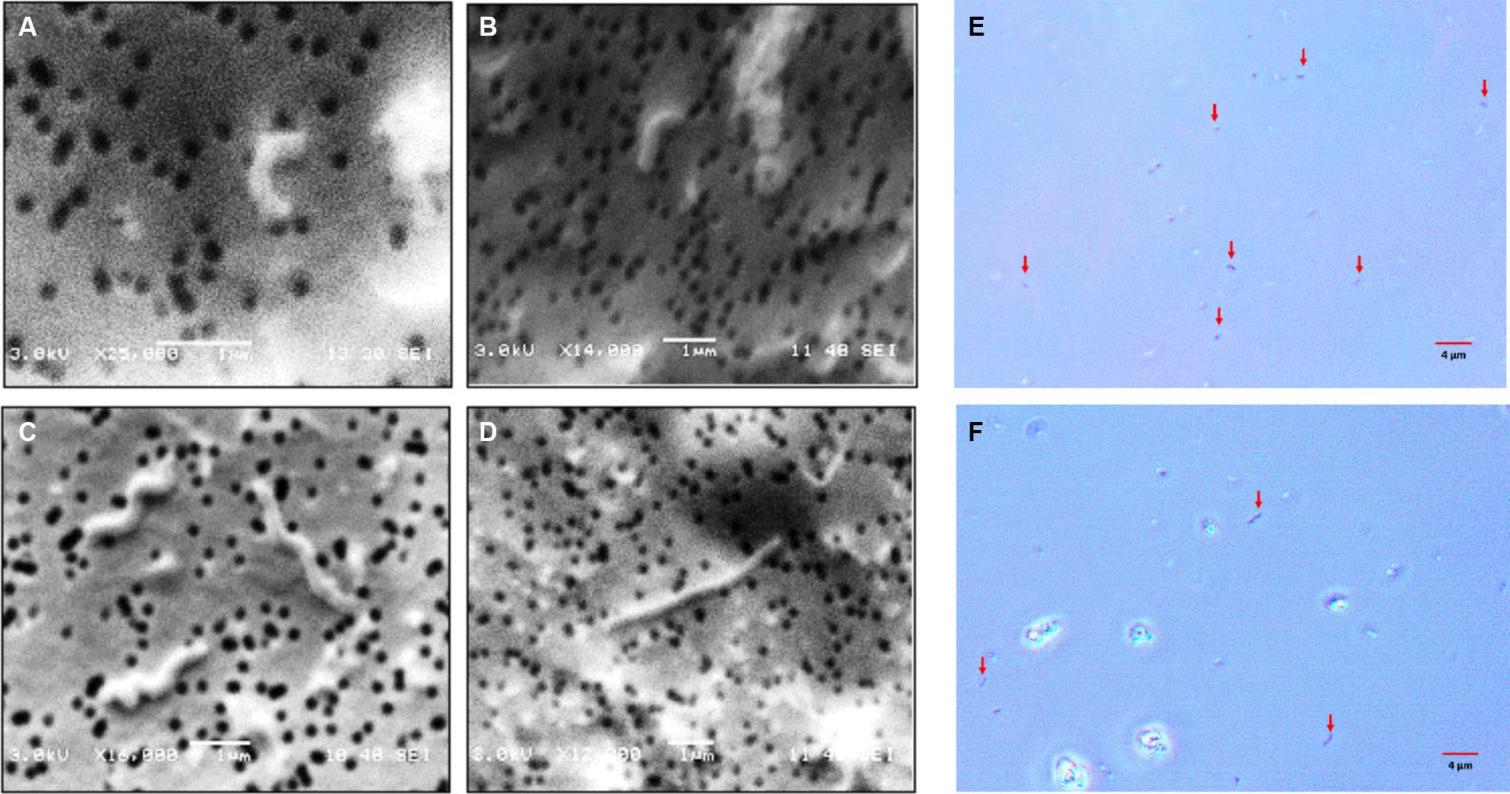
Microphotographs obtained by scanning electron and contrast phase microscopy of *L. ferriphilum* IESL25 cultures grown at different sulfate concentrations, **A,B,E** grown at 8 g/L and **C,D,F** at 80 g/L of sulfate (red arrows indicate cells in contrast phase microphotographs).

As observed in the profiles, the total cells numbers at the end of the growth experiments decreased, as higher concentrations of sulfate were included in the culture medium (Figure 2). On the other hand, in the control condition (8 g/L), the cells showed average sizes of approximately 1 μm, meanwhile, in cultures growth at higher sulfate concentration (80 g/L), the average cell size was approximately 4 μm. These size differences were observed by contrast phase and scanning electron microscopy (SEM) (Figure 3).

Obtaining cells from the different conditions, at the same exponential growth phase point allows performing proteomic analyses of cells at similar physiological phase, to ensure the reproducibility of the results and validating the comparison of the effect of different concentrations of sulfate (Matthiesen and Bunkenborg, 2020). Previous studies have suggested that cells in the stationary growth phase are structurally, physiologically, and functionally different from those in the exponential growth phase (Bathke et al., 2019). However, it has been shown that genes expressed in response to osmotic shock could also be involved in adaptation to the stationary phase, thus when cells enter the stationary phase, the general metabolism is reorganized and reserve compounds such as polyphosphates accumulate and glycogen, in addition to osmolytes such as trehalose, glycine betaine, and other compatible solutes (Jakowec et al., 2023). The latter justifies the choice for cell collection point, during the exponential phase growth and the importance of not exceeding the time for cell collection in order to avoid false positive results in proteomic analyses. It should also be noted that at the chosen collection time, the ferrous ion is still present in the solution, which ensures that cells have a source of energy and are still growing in such a way that the risk of the expression of related proteins and other types of stress are minimized.

A relevant change observed in *L. ferriphilum* IESL25 cells from a culture grown in the presence of 80 g/L of sulfate was the increase in the length of the cells by approximately 4-fold, compared to the control culture. Morphological changes seek to maintain the consistency of the cell under extreme conditions, such as increased osmotic pressure, and in this case, environmental stress due to elevated sulfate concentration. Perhaps the most frequent change resulting from environmental stress is filamentation (Nepal and Kumar, 2018). Filamentation of *L. ferriphilum* in response to sulfate could be a strategy that enables bacteria to survive in challenging environmental conditions by temporarily halting cell division and elongating their shape. This adaptation would allow them to better handle osmotic imbalances until conditions become more favourable for optimal growth and cell division (Nepal and Kumar, 2020).

### 3.4 Identification of compatible solutes in response to magnesium sulfate stress

**Figure 4:**
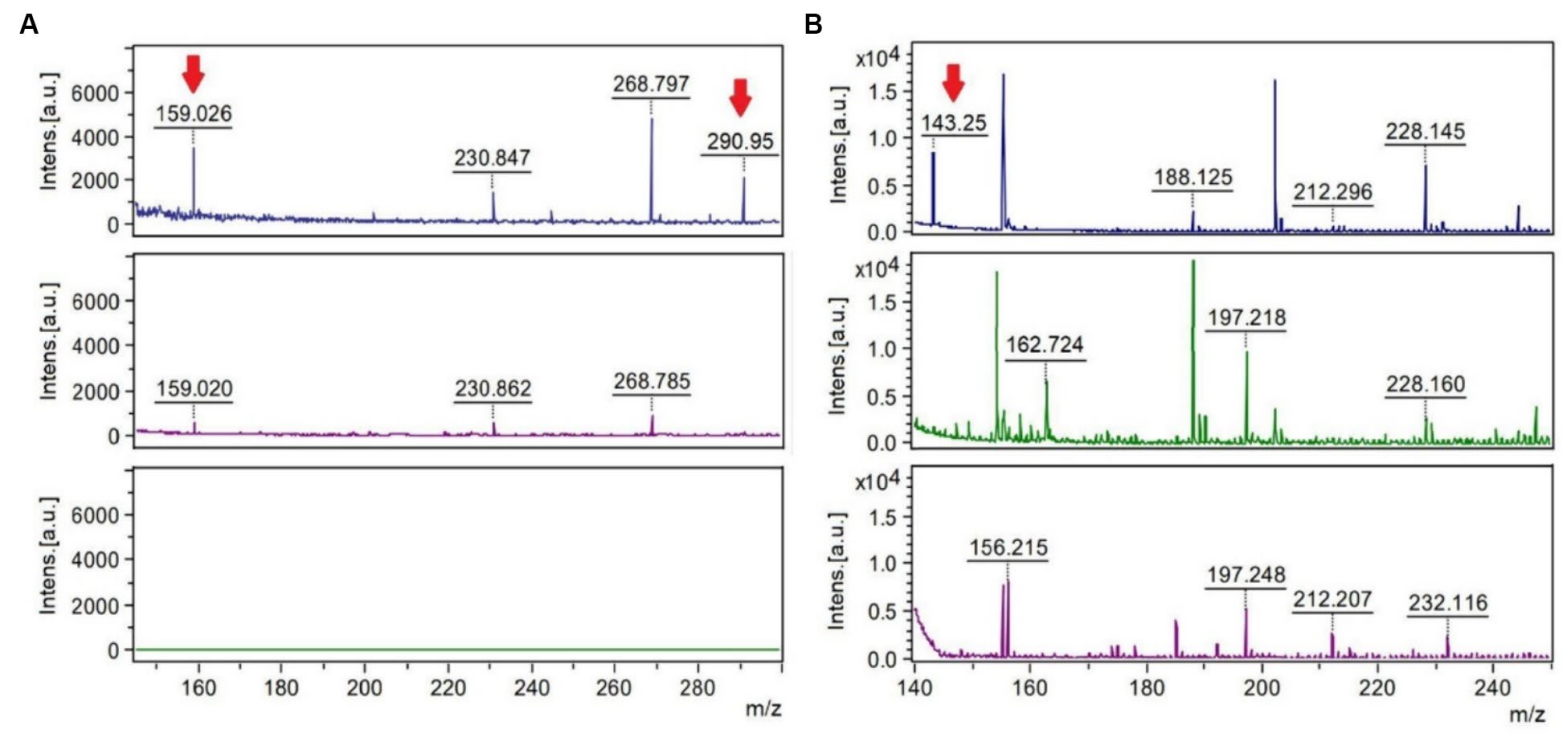
MALDI-TOF spectra using the stainless-steel matrix with TiO_2_^+^ CsI molecular weight peaks of 150–300 Da **(A)** and the NALDI^+^ CsI matrix with molecular weight peaks of 140–250 **(B)**. The upper spectra represent the condition of 80 g/L of sulfate, the central spectra correspond to 8 g/L of sulfate (control), and the lower spectra correspond to the control of the matrix or blank. The mass/charge (m/z) peaks indicated with the red arrow coincide with the adducts (a chemical compound that arises from the direct combination of two chemical species) Hydroxyectoine-H^+^ (159.026), Hydroxyectoine-Cs^+^ (290.95) and Ectoine-H^+^ (143.25).

MALDI-TOF analyses after hypo-osmotic shock approach showed peaks of mass/charge ratios similar to molecular weights of ectoine and hydroxyectoine as indicated in Figure 4. Similar findings were observed using two different ionization matrixes. For TiO_2_^+^ CsI matrix (Figure 4A), Hydroxyectoine (H^+^ adducts) has a fold change (treated/control) of 6.17 and Hydroxyectoine-Cs^+^ a fold change of 10.41. For NALDI^+^ CsI matrix (Figure 4B), Ectoine (H^+^) has a fold change of 8.75.

During the bioleaching process of low-grade copper ore and the generation of AMD, microorganisms are subjected to different environmental stressing conditions, such as changes in temperature, pH, concentration of sulfate, toxic metals, and lack of nutrients, among others (Jia et al., 2019). In response to some of these environmental changes, specifically, salt stress, bacteria modify gene expression or the production and accumulation of compatible solutes, also known as osmoprotectants or osmolytes (Gumulya et al., 2018). Here, two osmolytes ectoine and hydroxyectoine were identified. Because the matrixes used to carry out this were TiO_2_^+^ CsI and NALDI^+^ CsI, it must be emphasized that the detection of peaks and their intensities depend on the ionizability of the compounds in the matrix used. Regarding the spectra obtained by using each matrix, it was observed that adduct peaks associated with the molecular weight of hydroxyectoine were detected by using TiO_2_ as a matrix, in addition to perceiving fewer background signals (blank) than with the NALDI matrix. Despite the latter, NALDI matrix allowed the detection of several entities with intense signals, including a signal associated with the molecular weight of the osmolyte ectoine. Ectoine and hydroxyectoine act as compatible solutes by stabilizing cellular structures, enzymes, and macromolecules, preventing their denaturation, and ensuring cell survival in extreme environments (Corbett et al., 2022). Growing *L. ferriphilum* IESL25 requires a minimal number of total salts for its development. In the basic salt-based media used, no organic substances or osmolyte precursors were supplemented. Therefore, the identification of these osmolytes suggests the production of these compounds from scratch in response to environmental challenges such as high sulfate concentration. There have been only a limited number of studies conducted on microorganisms with acid and salt tolerance and most of it is related to the NaCl stress (Gumulya et al., 2018). Ectoine accumulation has been shown to increase halotolerance in *Leptospirillum* and *Acidihalobacter species* (Khaleque et al., 2019). Comparative genomic analyses have suggested trehalose as another potential compatible solute produced by *Leptospirillum* spp. (Rivera-Araya et al., 2020) and it has been previously detected in cultures of *Leptospirillum ferriphilum* grown in the presence of elevated concentrations of sulfate (Galleguillos et al., 2018).

### 3.5 2-DE protein profiles

**Figure 5:**
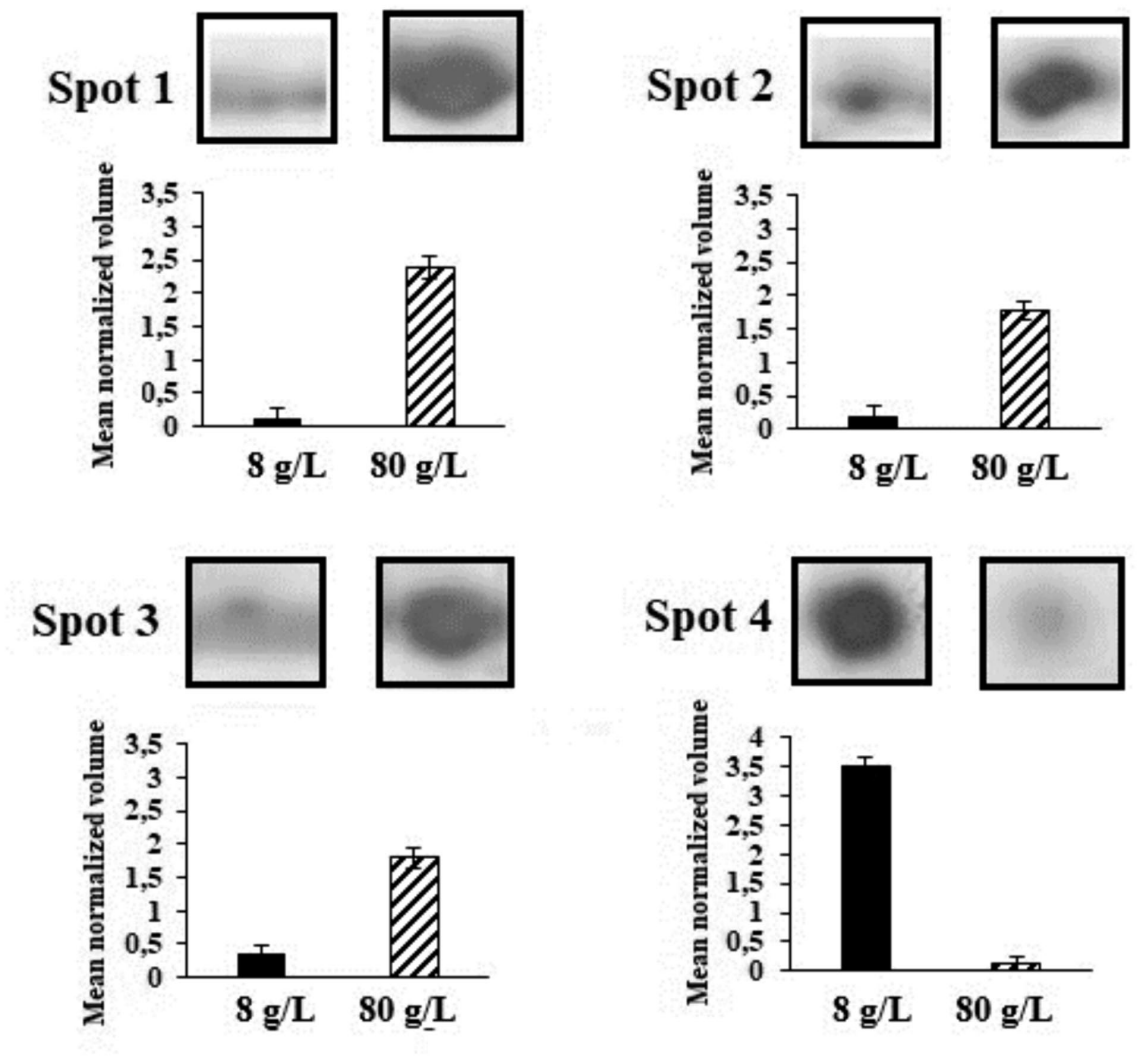
Spots in 2-DE gels in the pH range 5-8NL grown under the control condition of 8 g/L of sulfate and with 80 g/L of sulfate. Spots 1 to 4 represent the proteins differentially expressed and identified by LC-MS/MS.

Two-dimension protein electrophoresis gels (2-DE) in the pH range 5-8NL of protein extracts obtained from cultures grown under the control condition of 8 g/L and 80 g/L of sulfate are represented in Figure 5 (full gel images in Supplementary Figure S1). These protein electrophoresis assays were performed by triplicate and the expression analyses were subsequently carried out in accordance with point 2.5 of Materials and Methods section. Spots 1 to 4 represent the proteins differentially expressed and identified by LC-MS/MS. The mean normalized volume of spot 1 was 20.65 ± 0.18; spot 2, 9.59 ± 0.18; spot 3, 5.20 ± 0.15, and spot 4, 0.03 ± 0.02 (Supplementary Table S1).

The protein prediction results were based on the analyses of peptides obtained for each excised gel spot (protein). Table 1 shows the BLASTp analyses for each peptide obtained from gel spots sorted from highest to lowest score. In all cases, the peptides corresponded to the same protein in different species of the genus *Leptospirillum.*

**Table 1:** Summary and properties of the predicted proteins of *L ferriphilum* IESL25.

Spot	Protein	Accession No.	Functional category	pI; Mol. Wt (Da)^a^	Amino acids^a^	Score	Coverage %	*e*-Value
1	Isocitrate dehydrogenase (NADP) (IDH)	WP_036082354.1	Part of the TCA cycle R-TCA	6.72/36,600	336	78.3	100	8 × 10^−15^
2	Succinyl-CoA synthetase subunit alpha (SCoA)	WP_014960562.1	Part of TCA cycle and R-TCA	6.55/34,561	328	53.7	100	1 × 10^−6^
3	Aspartate-semialdehyde dehydrogenase (ASD)	WP_053765121.1	Oxidoreductase	6.8/37,615	349	96.1	100	2 × 10^−20^
4	Heat shock protein Hsp20/alpha crystallin family protein (Hsp20)	WP_014961909.1	Molecular chaperone	5.76/19,167	167	45.6	100	5 × 10^−4^

a Values given by Compute pI/Mw from the Expasy database.

Proteomic investigations of *Acidihalobacter aeolianus* DSM 14174^T^, indicated a remarkable 422-fold increase in ectoine synthase expression under conditions of elevated salt stress (Khaleque et al., 2018). Rivera-Araya et al. (2019) concluded that chloride stress up-regulated genes for the synthesis of potassium transporters (kdpC and kdpD), and biosynthesis of the compatible solutes (hydroxy)ectoine (ectC and ectD) and trehalose (otsB). On the other hand, *Acidihalobacter prosperus* DSM 5130, *A. prosperus* strain F5, *A. prosperus* strain V6, and *A. ferrooxidans* strain V8 represent a group of halotolerant, iron- and sulfur-oxidizing acidophiles whose genomes have been sequenced. The analysis of their genomes revealed the presence of genes associated with the production of osmoprotectants like ectoine and proline, as well as genes responsible for synthesizing periplasmic glucans and osmolyte transporters (Ossandon et al., 2014; Khaleque et al., 2017a,b,c).

This background is closely related to the differentially identified proteins represented in Figure 5. The first protein of interest corresponds to Aspartate-semialdehyde dehydrogenase (ASD). ASD is an enzyme that plays a crucial role in the biosynthesis of certain amino acids and compatible solutes in microorganisms. It is highlighted that one of the key functions of ASD involves the production of the compatible solute ectoine (Becker and Wittmann, 2020). ASD catalyses the conversion of L-aspartate semialdehyde to L-aspartate-4-semialdehyde, which is further processed by other enzymes in the ectoine biosynthesis pathway. These enzymes convert L-aspartate-4-semialdehyde into ectoine and its precursor, N-γ-acetyl-L-2,4-diaminobutyric acid (Becker and Wittmann, 2020). Other enzymes that increased their expression correspond to Isocitrate dehydrogenase (IDH) and Succinyl-CoA synthetase (SCoA). The relationship between IDH, SCoA, and ectoine production lies in the fact that the TCA cycle, in which both enzymes produce metabolites that can be utilized as precursors or intermediates in the biosynthesis of ectoine. During cellular stress TCA cycle plays an important role by modulating NADH/NADPH homeostasis, scavenging reactive oxygen species, producing ATP by substrate-level phosphorylation, signalling, and supplying metabolites to cope with a wide range of cellular disruptions (MacLean et al., 2023). For instance, alpha-ketoglutarate, which is produced by the action of IDH, can serve as a precursor for ectoine biosynthesis. On the other hand, the energy generated in the TCA cycle by SCoA may also play a role in providing the necessary ATP or GTP for ectoine biosynthesis. Thus, the TCA cycle and its associated enzymes could indirectly induce the production of ectoine in *L. ferriphilum* (MacLean et al., 2023). Finally, according to expression analysis, Hsp20 protein was inhibited under osmotic stress by magnesium sulfate. A wide range of microorganisms, including extremophiles, have at least one homolog of Hsp. Izquierdo-Fiallo et al. (2023) analysed Hsp20-encoding genes are widely distributed and highly redundant in acidophiles, however, their low level of expression suggests that they are proteins not specialized to act under conditions of cellular stress. Although functions that help the correct folding of proteins are attributed, this does not mean that they are proteins that are always expressed in stress situations.

## 4 Conclusion

Based on the description provided, the following conclusionsemerge:

The presence of sulfate ion at elevated concentration exerts a significant influence in the growth of *L. ferriphilum* IESL25 and therefore in the efficiency of the industrial bioleaching process, where this microorganism is involved. In prokaryotes, several mechanisms have been described to cope with changes in osmolarity, nevertheless there have been few studies focused on the changes of protein expression and identification of potential compatible solutes, when bioleaching microorganisms are exposed to elevated concentrations of sulfate. In this regard, mass spectrometry analyses allowed us to identify compounds resembling compatible solutes, specifically ectoine and hydroxyectoine, which were determined in cultures exposed to sulfate stress. In addition, the detection of these compatible solutes aligns seamlessly with the heightened synthesis of three proteins determined by 2-DE, which stand out as being significantly overexpressed under stress conditions: SCoA, ICDH and ASD.

By other hand, sulfate ion at elevated concentration exerts a significant influence on the cell division process of *L. ferriphilum* IESL25, resulting in a noticeable delay compared to the growth in relatively low sulfate concentration condition. This delay is substantiated by a reduced number of cells in cultures and the observation of cell filamentation determined by SEM, when subjected to stress conditions. This phenomenon could be attributed to the inhibition of genes responsible for cell division, driving filamented cells to grow by replicating cellular material without undergoing division, as a consequence of osmotic stress.

Finally, this study sheds light on the intricate web of interactions between osmotic stress, cell division, and the metabolism of critical proteins and solutes in a microorganism directly isolated from an industrial bioleaching process, when confronted with the challenges posed by sulfate stress conditions.

## Data availability statement

The datasets presented in this study can be found in online repositories. The names of the repository/repositories and accession number(s) can be found at: NCBI HQ902070.

## Author contributions

DA: Conceptualization, Formal analysis, Investigation, Methodology, Writing – original draft, Writing – review & editing. VZ: Conceptualization, Investigation, Writing – original draft, Writing – review & editing. IN: Formal analysis, Investigation, Writing – original draft, Writing – review & editing. MS: Conceptualization, Investigation, Writing – original draft, Writing – review & editing. PG: Conceptualization, Formal analysis, Funding acquisition, Investigation, Resources, Supervision, Writing – original draft, Writing – review & editing.

## Footnotes

^1^http://www.ncbi.nlm.nih.gov/

^2^http://www.arb-home.de

^3^http://www.thermo.com

^4^http://www.proteomecenter.org/software.php

^5^http://web.expasy.org/compute_pi/
