# Supplementary material for: Osmotic response in Leptospirillum ferriphilum isolated from an industrial copper bioleaching environment to sulfate
Source: Front Microbiol. 2024 May 24;15:1369244. doi: 10.3389/fmicb.2024.1369244 (PMC11157003; doi:10.3389/fmicb.2024.1369244)
Supplement: Supplementary file 1 [file Presentation_1.pptx]

## Slide 1
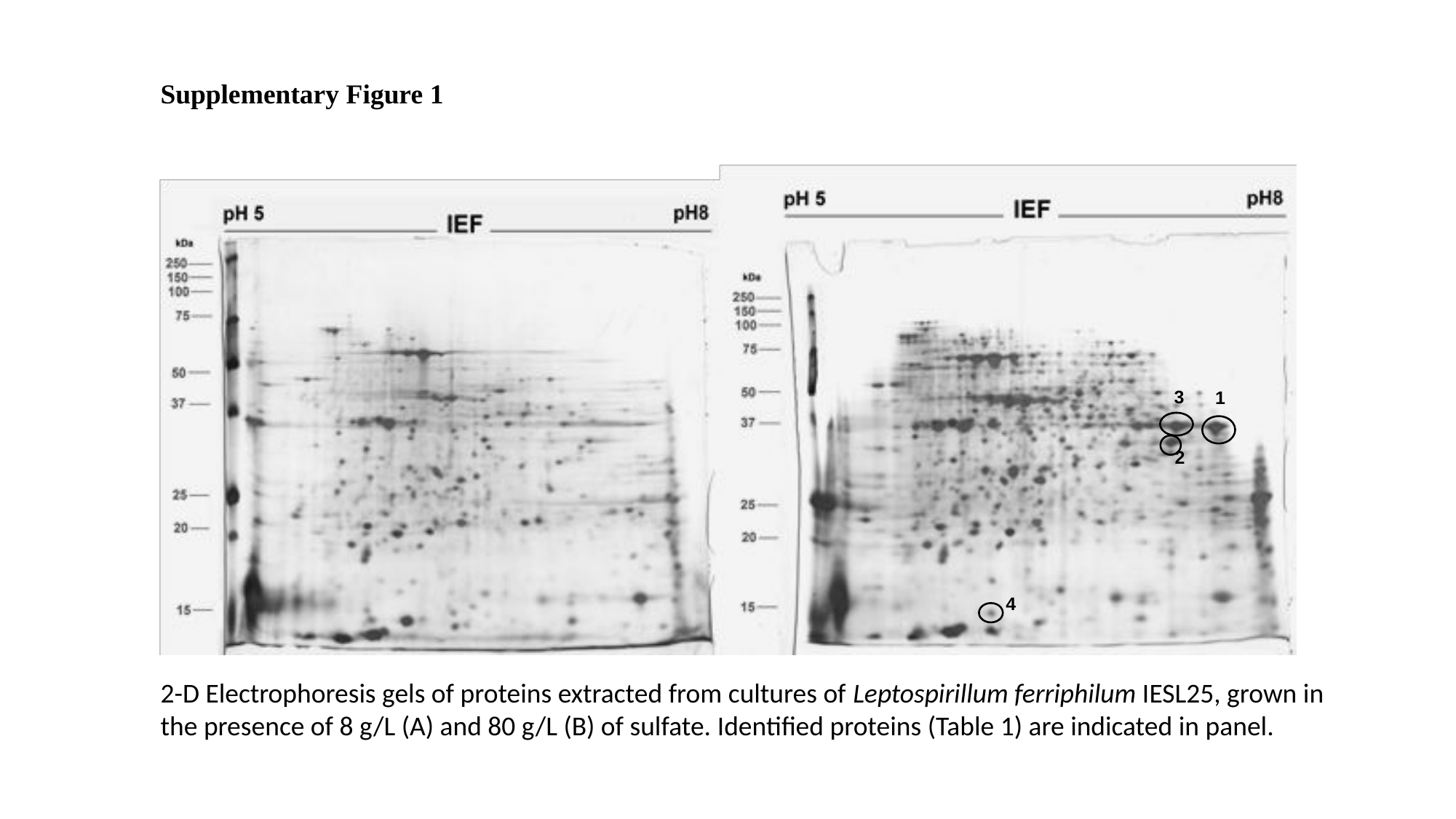

Supplementary Figure 1
3
1
2
4
2-D Electrophoresis gels of proteins extracted from cultures of Leptospirillum ferriphilum IESL25, grown in the presence of 8 g/L (A) and 80 g/L (B) of sulfate. Identified proteins (Table 1) are indicated in panel.

## Slide 2
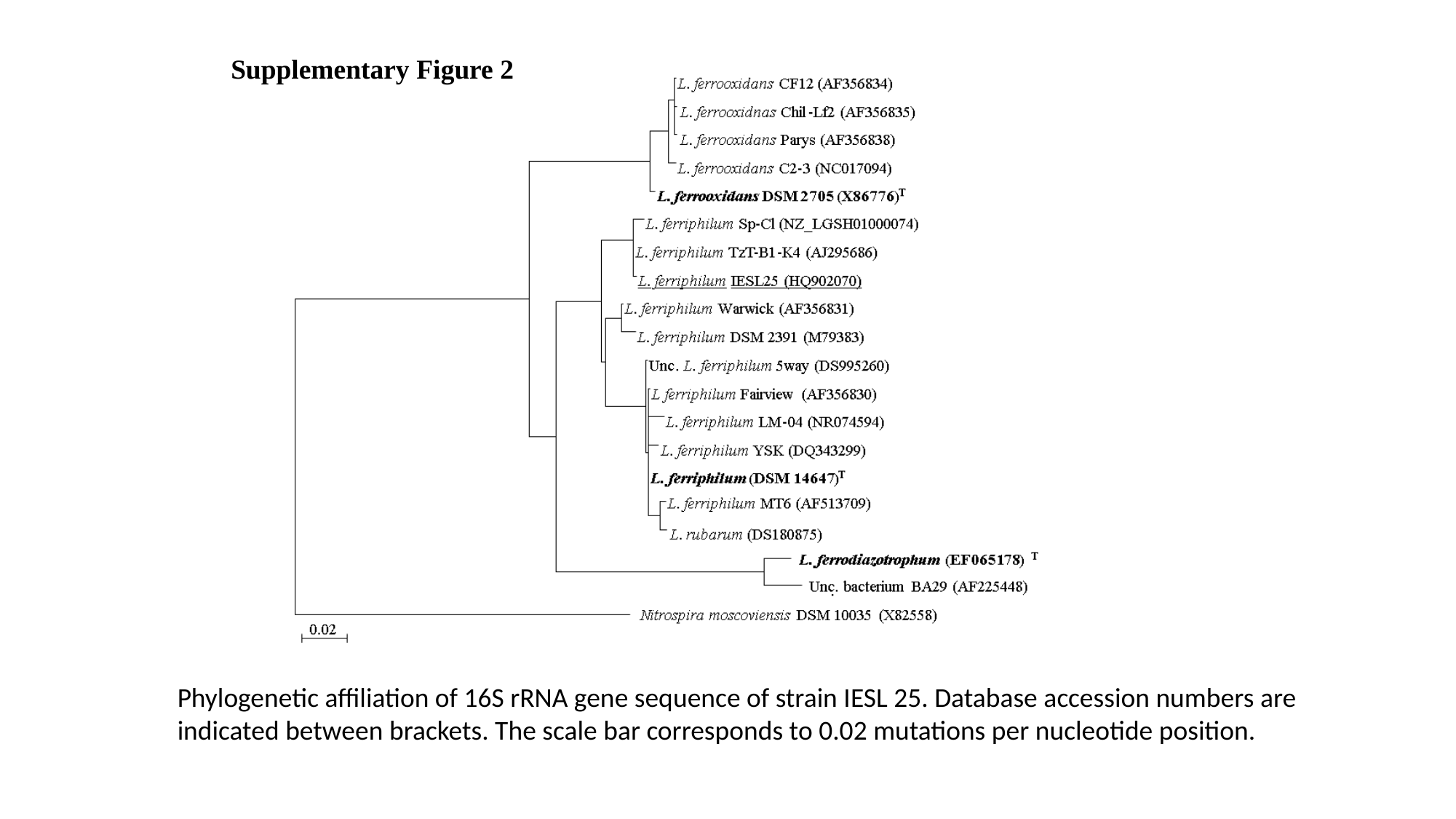

Supplementary Figure 2
Phylogenetic affiliation of 16S rRNA gene sequence of strain IESL 25. Database accession numbers are indicated between brackets. The scale bar corresponds to 0.02 mutations per nucleotide position.

## Slide 3
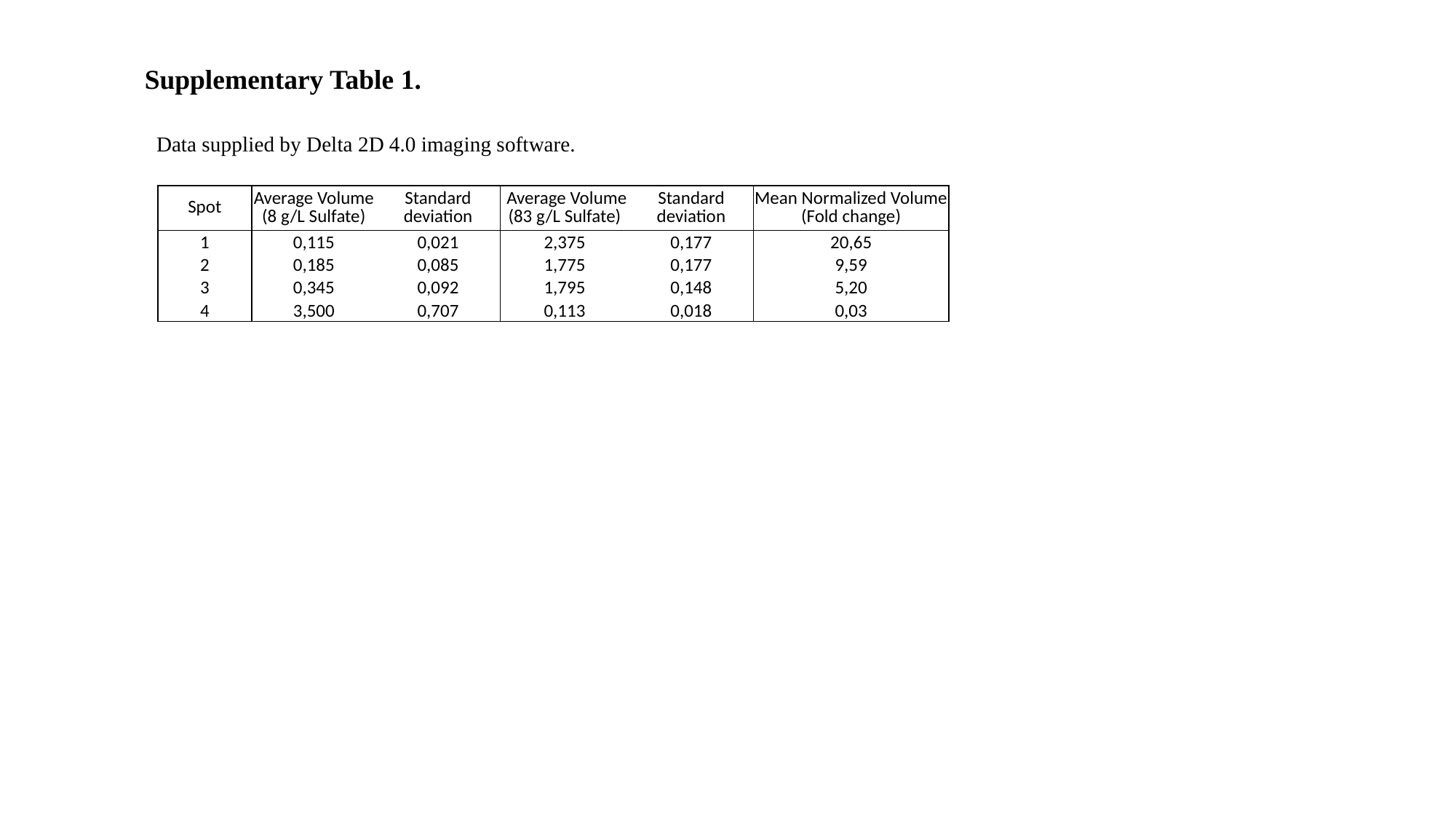

Supplementary Table 1.
Data supplied by Delta 2D 4.0 imaging software.
| Spot | Average Volume (8 g/L Sulfate) | Standard deviation | Average Volume (83 g/L Sulfate) | Standard deviation | Mean Normalized Volume (Fold change) |
| --- | --- | --- | --- | --- | --- |
| 1 | 0,115 | 0,021 | 2,375 | 0,177 | 20,65 |
| 2 | 0,185 | 0,085 | 1,775 | 0,177 | 9,59 |
| 3 | 0,345 | 0,092 | 1,795 | 0,148 | 5,20 |
| 4 | 3,500 | 0,707 | 0,113 | 0,018 | 0,03 |
